# Healthworker preparedness for COVID-19 management and implementation experiences: a mixed methods study in Uganda’s refugee-hosting districts

**DOI:** 10.1186/s13031-021-00415-z

**Published:** 2021-11-03

**Authors:** Gloria Seruwagi, Catherine Nakidde, Felix Otieno, Joshua Kayiwa, Brian Luswata, Eric Lugada, Eric Awich Ochen, Denis Muhangi, Betty Okot, Dunstan Ddamulira, Andrew Masaba, Stephen Lawoko

**Affiliations:** 1 Centre for Health and Social Economic Improvement (CHASE-i) - Department of Social Work and Social Administration (SWSA), Makerere University, Kampala, Uganda; 2 Department of Health Policy Planning and Management (HPPM), Makerere University School of Public Health (MakSPH), Kampala, Uganda; 3 Infotrak Research Consulting, Nairobi, Kenya; 4 Public Health Emergency Operations Centre (PHEOC), Ministry of Health, Kampala, Uganda; 5 Directorate of Health Governance and Regulation, Ministry of Health, Kampala, Uganda; 6 Agency for Cooperation in Research and Development (ACORD), Nairobi, Kenya; 7 The Lutheran World Federation (LWF), LWF, Kampala, Uganda; 8 Department of Public Health - Faculty of Medicine, Gulu University, Gulu, Uganda

**Keywords:** Health care workers (HCWs), Preparedness, Resilience, Health system, KAP, COVID-19

## Abstract

**Background:**

The negative impact of COVID-19 on population health outcomes raises critical questions on health system preparedness and resilience, especially in resource-limited settings. This study examined healthworker preparedness for COVID-19 management and implementation experiences in Uganda’s refugee-hosting districts.

**Methods:**

A cross sectional, mixed-method descriptive study in 17 health facilities in 7 districts from 4 major regions. Total sample size was 485 including > 370 health care workers (HCWs). HCW knowledge, attitude and practices (KAP) was assessed by using a pre-validated questionnaire. The quantitative data was processed and analysed using SPSS 26, and statistical significance assumed at *p* < 0.05 for all statistical tests. Bloom's cutoff of 80% was used to determine threshold for sufficient knowledge level and practices with scores classified as high (80.0–100.0%), average (60.0–79.0%) and low (≤ 59.0%). HCW implementation experiences and key stakeholder opinions were further explored qualitatively using interviews which were audio-recorded, coded and thematically analysed.

**Results:**

On average 71% of HCWs were knowledgeable on the various aspects of COVID-19, although there is a wide variation in knowledge. Awareness of symptoms ranked highest among 95% (*p* value < 0.0001) of HCWs while awareness of the criteria for intubation for COVID-19 patients ranked lowest with only 35% (*p* value < 0.0001). Variations were noted on falsehoods about COVID-19 causes, prevention and treatment across Central (*p* value < 0.0356) and West Nile (*p* value < 0.0161) regions. Protective practices include adequate ventilation, virtual meetings and HCW training. Deficient practices were around psychosocial and lifestyle support, remote working and contingency plans for HCW safety. The work environment has immensely changed with increased demands on the amount of work, skills and variation in nature of work. HCWs reported moderate control over their work environment but with a high level of support from supervisors (88%) and colleagues (93%).

**Conclusions:**

HCWs preparedness is inadequate in some aspects. Implementation of healthcare interventions is constrained by the complexity of Uganda’s health system design, top-down approach of the national response to COVID-19 and longstanding health system bottlenecks. We recommend continuous information sharing on COVID-19, a design review with capacity strengthening at all health facility levels and investing in community-facing strategies.

## Background

Globally, armed conflict remains a key humanitarian crisis that has led to significant forced displacement in the twenty-first century, greatly impacting on host country health systems [1]. With the emergence of the COVID-19 pandemic, significant challenges to health systems in general have been reported [2]. However, data on how the pandemic has impacted systems providing healthcare for refugee communities and the delivery of this care is scanty. This study scrutinizes the preparedness of health care workers (HCWs) for COVID-19 management in Uganda’s diverse refugee settings.

### Refugee hosting in Uganda

Uganda’s open-door approach to hosting refugees has received international acclaim with its refugee policy described as the most progressive [3] and “*the world’s most compassionate refugee policy*” [4]. The nation is among the world’s top three refugee-hosting nations and largest in Africa, currently hosting approximately 1.5 million refugees and asylum seekers, mostly from South Sudan. While refugees are spread out in 12 districts, over 67% live in the West Nile region [5]. Uniquely, Uganda has no refugee camps; instead refugees live in gazetted settlements or wherever they may choose within wider society [6]. This is in line with the government’s policy of service integration where refugees share all services with the host community [7]. Registered refugees have the freedom to freely move, be employed, engage in agriculture or business and access all services, including healthcare. While all refugee settlements in Uganda have lower-level health facilities, these facilities are also used by the local host communities integrated within or surrounding the settlements [8]. Health facilities in refugee settlements follow the national referral pathway which requires more complex cases from lower-level facilities to be managed at higher-level facilities [9]. How health systems have been affected by the COVID-19 pandemic has received some attention in the global literature [2]; however, the literature is scant on healthcare for refugee populations during this period.

### COVID-19 impact on health systems

The COVID-19 pandemic has crippled all systems across the globe, some more than others. Even health systems in the more sophisticated and relatively high-performing countries like the United Kingdom, Spain, Italy and United States have been severely affected [10]. Almost all health systems have faced challenges with getting enough personal protective equipment (PPE) and other essential supplies, shortages in health care workers (HCWs) and overflowing hospitals due to the pandemic [2, 11, 12]. While all the pillars of the health system have been greatly affected, several studies have also reported disruptions in both the physical and psychosocial wellbeing of HCWs due to infection with COVID-19, burnout or other COVID-19 related occupational stressors [13–20] and compromised working conditions as some of the greatest challenges faced by the health workforce [21]. Furthermore, the evidence has indicated that satisfactory knowledge about COVID-19 among HCWs was associated with positive attitudes towards treating COVID-19 in Cyprus, South Africa, Sierra Leone, Ethiopia and Nigeria [22–26].

In Uganda, the national response to COVID-19 included a lockdown and other preventive measures like banning of public gatherings, institutional closures and movement restrictions including a ban on public transport and curfew. These were grounded in the country’s previous efforts to curb outbreaks like Ebola and Marburg [27, 28]. The Ministry of Health (MOH) also instituted several guidelines for the continuity of essential health services and clinical management of COVID-19. This included screening, testing, isolation, institutional quarantine and setting up specific infection prevention and control (IPC) measures at health facility level. The World Health Organisation (WHO) played the critical role of developing guidance and training materials that could easily be adapted to local context by MOH and other COVID-19 responders [28]. However, several challenges continue to significantly undermine Uganda’s health system preparedness to tackle the pandemic. These include the high HCW-patient ratios exacerbated by existing staffing gaps, porous borders which occasionally let through infected people including undocumented refugees from neighbouring countries, ineffective quarantine processes and low financing for the health system [28–30].

COVID-19 is a highly contagious infectious disease with higher likelihood for infection among HCWs compared to the general population [31]. The evidence from developed countries with high incidence of COVID-19 shows that limitations in human and material capital can complicate its case management [32]. Additionally, the chronic shortages of PPEs alongside other IPC inputs have increased patient-to-staff transmissions with a heavy death toll on medical staff [33]. Consequently, these developments have caused concerns about the preparedness for COVID-19 management in struggling health systems. Moreover, previous research among HCWs has documented inadequate knowledge and distorted beliefs about causes, risk factors and treatments for different health conditions including cervical cancer [34], hypertension [35], diabetes [36] and mental illness [37]. It is therefore critical that HCWs have adequate knowledge about all aspects of COVID-19 from clinical manifestation, diagnosis, proposed treatment and prevention strategies [31].

While several studies have reported on health system preparedness including HCW knowledge, attitudes and practices (KAP) in sub-Saharan Africa and elsewhere in the world; to the best of our knowledge only one study [38] has reported on Ugandan HCWs. Even then, this study was conducted within four (4) university teaching hospitals, all located in the central region within approximately a 3–15 km radius from the National Referral Hospital (NRH) in Kampala, Uganda’s capital city. Our research builds on and complements the critical knowledge generated by this study to assess HCW perceptions on their knowledge and practice. We report on HCW’s perceived knowledge and implementation experiences with data collected in-person from a larger (> three-fold) sample size drawn from all levels of Uganda’s health system and different geographical regions. We also show implications of the national health system design on health outcomes for forcibly displaced populations in the pandemic era.

## Methods

### Study context: Uganda’s health care system

Uganda’s health system comprises of both the private and public sector in terms of infrastructure, ownership and delivery of health services [39, 40]. The health system is decentralized and hierarchical with seven levels starting from the household/village level and culminating at national referral institutions and the Ministry of Health [41]. In order of hierarchy there are the Village Health Teams (VHTs) or community health workers (CHWs) who are the first point of contact with health service users and resident within the village. Next is the Health Centre (HC) IIs, HCIIIs, HCIVs, District Hospitals, Regional Referral Hospitals (RRH) and National Referral Hospitals (NRH). In principle, each level of the health system is equipped to handle progressively complex cases while also referring to the next level upwards [9]. Referrals are therefore an integral part and key operational area of the success of the entire health system. The WHO lists the six key pillars or building blocks critical to any system’s functionality as service delivery, the health workforce, health information systems, access to essential medicines or vaccines, financing, and leadership or governance [42]. Having a hierarchical system also means that, across each one of the building blocks, lower-level units in Uganda can only handle less-complex cases which also determines the considerably less resources and inputs available for their use.

Majority of the health facilities are public and therefore government/donor funded where, ideally, services should be provided free of charge. However, the latest figures report private expenditure as a percentage of Current Health Expenditure (CHE) to be 41% and out-of-pocket expenditure at 38% [43]. Health financing remains a key national challenge and the recommended Abuja target 15% of the GDP apportioned to health [40, 44, 45] has never been achieved, with average health sector allocations ranging from 6 to 9% [45, 46]. In particular, the low funding for Uganda’s healthcare system continues to create an ongoing challenge of insufficient health workforce numbers [47] which has sometimes been mitigated by task shifting since 1918 [48] and which the evidence shows to have improved quality of care in the treatment of HIV/AIDS, tuberculosis, maternal, newborn and child health (MNCH) programs as well as malaria [49–51]. However, task shifting has the potential to significantly undo its intended benefits in the absence of adequate training or supervision from skilled health workers, inappropriate compensation and work overloads as is the case for Uganda [52]. Older and more recent assessments show that Uganda continues to experience serious shortfalls and challenges across all six of the building blocks in its health system [53, 54].

## Study design

This was a cross-sectional descriptive study using mixed-methods including a survey, key informant interviews (KII) and indepth interviews (IDI). The study’s primary population were health care workers at facilities serving refugee populations in both urban and rural settlements located in the Central, South Western, West Nile and Northern regions of Uganda. Secondary participants included key informants from government ministries and local government offices involved in the refugee response, local leaders, NGOs and international agencies such as UNHCR. We collected data from seventeen (17) health facilities located within refugee settlements and outside hospitals which were direct referral points.

### Sample size and sampling procedures

The infectious nature of COVID-19 and how quickly it is spread implies that all categories of HCWs are at risk, ranging from the essential frontline workers to administrators, as they all share the same healthcare environment with the patients. Therefore the inclusion criteria was all workers at risk of contracting COVID-19 in the healthcare facility, but selected with Probability Proportional to Size (PPS), i.e. their numbers at the healthcare setting. The total sample size required for the quantitative arm of the study (370 HCWs) was determined using Kish’s method for cross-sectional studies (formula 1 below) with the assumption that the proportion unprepared for COVID-19 management is previously unknown (i.e. in such cases, a proportion of 50% is usually assumed to ensure the largest possible sample, all other factors constant); a 95% confidence interval and a 5% margin of error. After the sample size of 370 was determined using Kish’s formula we then apportioned each HCW cadre their number according to their fraction of representation at the health unit.

1 $$n = z^{2} *p\left( {1 - p} \right)/e^{2}$$

where n is the optimal sample size, Z is the standard error of the confidence level (which is 1.96 if 95% confidence interval is applied), p is the proportion with the phenomena of interest (i.e. proportion prepared for COVID-19 management). We usually get this from previous studies. If no such previous study, take p = 0.5 as this guarantees the largest possible sample, all other factors constant, e is the margin of error, usually assumed between 1 and 5% for conservative error.

For the qualitative arm of the study, 115 key informants with representation from key HCW cadre groups were purposively selected for interviews and focus groups discussions. The break-down of specific numbers within each cadre group are presented in Table 1.

**Table 1:** Socio-demographic characteristics

Characteristic	n	%
*District*		
Adjumani	62	16.8
Gulu	45	12.2
Kabarole	55	14.9
Kampala	130	35.1
Kyegegwa	37	10.0
Mubende	41	11.1
*Region*		
Central	171	46.2
West Nile	69	18.7
Western	92	24.9
Northern	38	10.3
*Health facility level*		
Region Referral hospital	200	54.1
General hospital	70	18.9
HC IV	49	13.2
HC III	26	7.0
HC II	9	2.4
Clinic	9	2.4
*Facility type*		
Public	297	80.3
Private Not for Profit	73	19.7
*Gender*		
Male	162	43.8
Female	208	56.2
*Education*		
Primary	1	0.3
Secondary	2	0.5
Certificate/	83	22.4
Diploma	164	44.3
Vocational	2	0.5
Bachelors’ degree	103	27.8
*Marital status*		
Single	109	29.5
Married	241	65.1
Divorced/separated	10	2.7
Widowed	4	1.1
*Religion*		
Catholic	151	40.8
Protestant	126	34.1
Muslim	26	7.0
Pentecostal	55	14.9
SDA	12	3.2
*Cadre*		
Consultant	7	1.9
Medical officer	28	7.6
Clinical officer	38	10.3
Reg trained nurse	89	24.1
Enrolled nurse	87	23.5
Pharmacist	26	7.0
Radiographer	5	1.4
Laboratory technologist	32	8.6
Other	58	15.7
*Department*		
Emergency	13	3.5
Outpatient	160	43.2
Inpatient	125	33.8
Investigative dept	12	3.2
Other	60	16.2
*Age (Mean; SD)*	34.5 (7.7)	
*Number of Beds (Mean; SD)*	306 (446)	

## Measures

### Dependent variables

Knowledge, attitudes and practices (KAP) were assessed quantitatively using questionnaires capturing KAP in terms of causes, symptoms, risk factors, treatment and management of COVID-19. These were expressed in terms of statements with response alternatives on a Likert scale ranging between 1 and 4 i.e. “strongly disagree”, “disagree”, “agree”, “strongly agree”. Individual sums on each sub-scale (i.e. Knowledge, Attitude and Practice) were calculated, with higher scores representing higher KAP respectively. A modified Bloom's cutoff of 80% was used to determine threshold for sufficient knowledge level and practices on the various attributes measured [55]. The statements are presented in Tables 3 and 5, and comparison of scores across different groups in Table 4.

### Independent variables

Demographic, and healthcare related characteristics of HCW were collected using survey instruments. These included gender, marital status, education level, cadre, facility, department among others.

Qualitatively, KAP was assessed using apriori developed interview guides. The survey was conducted using both Computer Assisted Personal Interviews (CAPI) while qualitative methods applied both virtual and in-person interviews.

### Analysis

Qualitative interviews were audio-recorded, transcribed, coded and analysed thematically, aligning with study objectives. The quantitative data was processed and analyzed using SPSS 26, with statistical significance assumed at *p* < 0.05 for all tests. Knowledge, attitude and practices were compared across regions, health facility levels, facility types, gender and levels of education using chi-square tests and t-tests.

## Results

### Sociodemographic characteristics

A sample of health care workers (n = 370) were recruited from seventeen (17) health facilities serving refugees and host communities in seven (7) districts of Uganda. These health facilities are located in the Central, Western, Northern and North Western (West Nile) regions which also host refugees of different nationalities. Majority of the HCWs worked in regional referral and general hospitals, private facilities and held a diploma or certificate education in a medical field. There were slightly more women than men, and Catholics or Protestants were dominant. Participants varied in cadre, ranging from medical specialist to laboratory technologist; and were mainly placed in outpatient and inpatient settings. There was large variation in number of beds between the different health facilities owing to their variation in facility size; however, on average the facilities studied had 302 beds. The average age in the studied sample was 35 years, as shown in Table 1 below:

### Knowledge

One of this study’s objectives was to assess the self-reported knowledge, attitudes and practices (KAP) of healthcare workers in the pandemic era. Participants’ overall KAP was analyzed using the sum score of each outcome and categorized Bloom's cut-off point [55].

Generally, there was a high level of knowledge among the HCWs on symptoms of COVID-19 (95%), behavioral risk factors for COVID-19 transmission (93%), that COVID-19 mortality is heightened if patient has other health conditions (92%), which patient needs to be tested for COVID-19 (89%), behavioral risk factors for COVID-19 progression (88%), what PPE to use in the fight against COVID-19 (86%), demographic risk groups for COVID-19 transmission (83%), detailed clinical information about COVID-19 (82%) and demographic risk groups for COVID-19 mortality (80%).

We found average knowledge on myths about causes and prevention of COVID-19 (78%), falsehoods about causes, prevention and treatment of COVID-19 (76%), hospital’s criteria for admission (75%), terminology of the virus causing COVID-19 (74%), myths about causes, prevention and treatment of COVID-19 (74%), readiness to manage COVID-19 as a health worker (69%), case definition of COVID-19 in use in Uganda (67%), type of testing necessary for COVID-19 (66%) and how to properly don or doff the full PPE for COVID-19 care (64%).

**Fig. 1:**
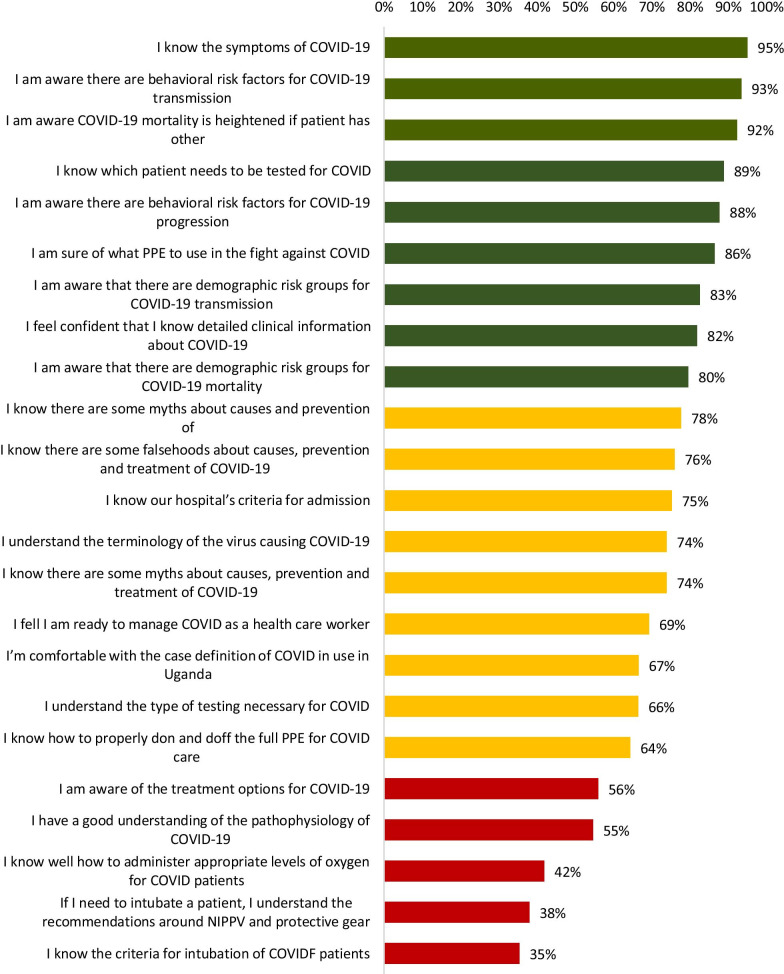
Knowledge levels of health workers regarding the management of COVID-19

There were low levels of knowledge on treatment options for COVID-19 (56%), understanding of the pathophysiology of COVID-19 (55%), how to administer appropriate levels of oxygen for COVID-19 patients (42%), the recommendations around NIPPV and protective gear (38%), criteria for intubation of COVID patients (35%). The information is presented in Fig. 1 below.

There was no significant variation in knowledge levels across the 17 facilities in the surveyed regions. There was, however, a significantly high proportion (*p* value = 0.0356) of HCWs with knowledge on falsehoods about causes, prevention and treatment of COVID-19 in Central compared to the other regions. There was also a higher proportion (*p* value = 0.0083) of HCWs aware of knowledge on treatment options for COVID-19 in Central compared to the other regions. There was a significantly higher proportion (*p* value = 0.0349) of HCWs with knowledge on how to properly don and doff the full PPE for COVID care in South Western region compared to the other regions. The information is shown in Table 2 below:

**Table 2:** Variations in knowledge about COVID-19 among health workers by region

	Central (n = 171) (%)	*P* value	West Nile (n = 69) (%)	*P* value	South Western (n = 35) (%)	*P* value	Northern (n = 95) (%)	*P value*	Total (n = 370) (%)
I know the symptoms of COVID-19	93	0.1455	97	0.0084	97	0.6618	97	0.4066	95
I am aware there are behavioral risk factors for COVID-19 transmission	93	0.3499	97	0.4742	94	0.6033	91	0.5072	93
I am aware COVID-19 mortality is heightened if patient has other	94	1.0000	94	0.2155	86	0.8262	90	0.5314	92
I know which patient needs to be tested for COVID	86	0.4090	88	0.5702	91	0.2311	94	0.1474	89
I am aware there are behavioral risk factors for COVID-19 progression	91	0.3189	87	0.8102	89	0.7200	83	0.1969	88
I am sure of what PPE to use in the fight against COVID	84	0.3021	80	0.8170	97	0.8635	92	0.1185	86
I am aware that there are demographic risk groups for COVID-19 transmission	86	0.5419	74	0.2031	80	0.0690	84	0.8163	83
I feel confident that I know detailed clinical information about COVID-19	87	0.3782	68	0.0791	85	0.6583	83	0.8204	82
I am aware that there are demographic risk groups for COVID-19 mortality	83	0.4101	75	0.3514	77	0.6776	78	0.6665	80
I know there are some myths about causes and prevention of	80	0.5988	78	1.0000	80	0.7874	72	0.2172	78
I know there are some falsehoods about causes, prevention and treatment of COVID-19	84	0.0356*	62	0.0161*	74	0.7947	72	0.4213	76
I know our hospital’s criteria for admission	68	0.0900	79	0.4811	77	0.7965	84	0.0639	75
I understand the terminology of the virus causing COVID-19	78	0.3175	67	0.2337	86	0.1226	67	0.1730	74
I know there are some myths about causes, prevention and treatment of COVID-19	78	0.3175	74	1.0000	63	0.1673	70	0.4329	74
I fell I am ready to manage COVID as a health care worker	70	0.8152	58	0.0763	77	0.3323	74	0.3432	69
I’m comfortable with the case definition of COVID in use in Uganda	70	0.4882	59	0.2024	83	0.0553	58	0.1009	67
I understand the type of testing necessary for COVID	68	0.6475	68	0.7490	54	0.1612	67	0.8543	66
I know how to properly don and doff the full PPE for COVID care	64	1.0000	57	0.2732	82	0.0349*	65	0.8562	64
I am aware of the treatment options for COVID-19	68	0.0083*	43	0.0486*	57	0.9106	43	0.0236*	56
I have a good understanding of the pathophysiology of COVID-19	62	0.1272	39	0.0154	71	0.0723	46	0.1172	55
I know well how to administer appropriate levels of oxygen for COVID patients	47	0.0090*	35	0.2260	34	0.2648	44	0.1179	42
If I need to intubate a patient, I understand the recommendations around NIPPV and protective gear	41	0.2770	29	0.2816	49	0.3655	41	0.8602	38
I know the criteria for intubation of COVIDF patients	39	0.5072	27	0.1577	34	0.2089	35	0.5902	35

Across health facilities, there was a significantly lower proportion of HCWs in general hospitals with awareness on detailed clinical information about COVID-19 (*p* value = 0.0077), falsehoods about causes, prevention and treatment of COVID-19 (*p* value = 0.0092), terminology of the virus causing COVID-19 (*p* value = 0.0406), readiness to manage COVID-19 (*p* value = 0.0095), treatment options for COVID-19 (*p* value = 0.0022), understanding of the pathophysiology of COVID-19, (*p* value = 0.0215). Lower proportion (*p* value = 0.007) of HCWs in HCIV were aware of COVID-19 mortality being heightened if a patient has other health conditions. Awareness on how to administer appropriate levels of oxygen for COVID patients was significantly higher (*p* value = 0.041) in referral hospitals compared to other facility levels. This information is further shown in Table 3 below:

**Table 3:** Variations in perceived knowledge about COVID-19 among HCWs by facility level

	Referral Hospital (%)	*P* value	General Hospital (%)	*P* value	HC IV (%)	*P* value	Total (%)
I know the symptoms of COVID-19	96	0.5914	97	0.4684	90	0.1531	95
I am aware there are behavioral risk factors for COVID-19 transmission	91	0.3966	94	0.7617	98	0.1801	93
I am aware COVID-19 mortality is heightened if patient has other conditions	95	0.1824	91	0.7798	80	0.007*	92
I know which patient needs to be tested for COVID	89	1.000	91	0.6201	84	0.3052	89
I am aware there are behavioral risk factors for COVID-19 progression	88	1.000	84	0.3569	90	0.6836	88
I am sure of what PPE to use in the fight against COVID-19	86	1.000	80	0.1979	88	0.7031	86
I am aware that there are demographic risk groups for COVID-19 transmission	84	0.762	77	0.2315	78	0.3886	83
I feel confident that I know detailed clinical information about COVID-19	87	0.126	68	0.0077*	78	0.4986	82
I am aware that there are demographic risk groups for COVID-19 mortality	81	0.776	76	0.4493	80	1.000	80
I know there are some myths about causes and prevention of COVID-19	78	1.000	73	0.3616	78	1.000	78
I know there are some falsehoods about causes, prevention and treatment of COVID-19	80	0.280	61	0.0092*	78	0.7577	76
I know our hospital’s criteria for admission	81	0.107	78	0.5935	63	0.0740	75
I understand the terminology of the virus causing COVID-19	76	0.603	62	0.0406*	69	0.4575	74
I know there are some myths about causes, prevention and treatment of COVID-19	76	0.603	70	0.4885	71	0.6549	74
I fell I am ready to manage COVID as a health care worker	76	0.080	53	0.0095*	64	0.4801	69
I’m comfortable with the case definition of COVID in use in Uganda	68	0.810	58	0.1471	65	0.7805	67
I understand the type of testing necessary for COVID	68	0.632	64	0.7471	58	0.2707	66
I know how to properly don and doff the full PPE for COVID care	63	0.814	53	0.0824	65	0.8911	64
I am aware of the treatment options for COVID-19	62	0.169	36	0.0022*	58	0.7913	56
I have a good understanding of the pathophysiology of COVID-19	57	0.649	40	0.0215*	59	0.5973	55
I know well how to administer appropriate levels of oxygen for COVID patients	51	0.041*	31	0.0859	37	0.5052	42
If I need to intubate a patient, I understand the recommendations around NIPPV and protective gear	43	0.248	30	0.2039	38	1.0000	38
I know the criteria for intubation of COVID-19 patients	43	0.062	26	0.1445	25	0.1652	35

### Bivariate analyses on perceived knowledge and awareness

*Geographical*: HCWs ratings of their knowledge varied geographically. At district level, HCWs in Gulu and Kyegegwa rated their overall knowledge and case-management skills significantly higher than colleagues in other districts. With regard to *facility* variations, healthworkers at HC III and Regional Referral hospitals rated their overall knowledge and case management-related skills significantly higher than HCW at other levels on average. In terms of *department or position* variations, consultants and medical officers demonstrated knowledge in all regards higher than other staff cadre groups on average. There was no significant variation in knowledge by *department or gender*. However, there was a significantly higher proportion (*p* value = 0.0239) of male HCWs who understood the terminology of the virus causing COVID-19 compared to female HCWs. Compared across levels of *education*, HCWs who had attained atleast a Bachelor’s degree were significantly knowledgeable on detailed clinical information about COVID-19 (*p* value = 0.0066), terminology of the virus causing COVID-19 (*p* value = 0.0028), treatment options for COVID-19 (*p* value = 0.0062), understanding of the pathophysiology of COVID-19 (*p* value = 0.0010) as compared to HCWs with other qualifications. HCWs with certificate level education reported significantly lower knowledge on understanding the terminology of the virus causing COVID-19 (*p* value = 0.0002), understanding the type of testing necessary for COVID-19 (*p* value = 0.0038), treatment options for COVID-19 (*p* value = 0.0051), pathophysiology of COVID-19 (*p* value = 0.0010), how to administer appropriate levels of oxygen for COVID-19 patients (*p* value = 0.0438). There was no significant variation in knowledge by age, marital status and religion of HCWs (Table 4).

**Table 4:** Variations in HCWs knowledge and awareness by socio-demographic and facility factors

Characteristic	Overall knowledge (24–96) Mean (SD)	Knowledge of relevance to case management (15–60) Mean (SD)	Knowledge of relevance to prevention (9–36) Mean (SD)
*District*			
Adjumani	65.8 (12.5)*	38.9 (8.8)*	26.9 (5.0)
Gulu	70.1 (12.9)	42.4 (8.9)	27.7 (5.1)
Kabarole	65.1 (10.9)	38.8 (6.9)	26.3 (5.1)
Kampala	68.7 (9.55)	40.8 (6.9)	27.9 (3.9)
Kyegegwa	70.3 (12.3)	43.2 (8.0)	27.1 (5.0)
Mubende	68.1 (12.3)	40.5 (9.0)	27.6 (4.3)
*Health facility level*			
Region Referral hospital	68.7 (11.2)*	41.2 (7.9)*	27.5 (4.5)
General hospital	65.1 (12.0)	38.3 (8.6)	26.7 (4.6)
HC IV	67.5 (12.0)	40.3 (8.3)	27.2 (4.9)
HC III	73.0 (10.6)	44.6 (6.1)	28.4 (5.4)
HC II	60.6 (8.3)	36.1 (6.4)	24.4 (2.5)
Clinic	66.2 (6.2)	38.4 (3.2)	27.8 (3.9)
*Gender*			
Male	69.4 (11.0)*	41.5 (7.7)*	27.9 (4.6)*
Female	66.7 (11.6)	39.8 (8.1)	26.6 (4.5)
*Education*			
Certificate	63.6 (11.7)*	37.8 (8.5)*	26.6 (4.2)*
Diploma	67.2 (10.7)	40.2 (7.5)	26.9 (4.5)
Bachelors’ degree	71.8 (10.9)	42.8 (7.5)	28.6 (4.7)
*Marital status*			
Single	68.8 (10.9)	41.0 (7.4)	27.8 (4.5)
Married	67.4 (11.5)	40.4 (8.1)	27.0 (4.6)
Divorced/separated	69.7 (7.9)	40.9 (6.7)	28.8 (2.3)
Widowed	74.0 (24.1)	44.3 (17.3)	29.8 (7.3)
*Religion*			
Catholic	67.9 (11.1)	40.6 (7.9)	27.3 (4.5)
Protestant	66.9 (11.6)	40.0 (7.9)	26.9 (4.6)
Muslim	70.5 (11.6)	42.8 (8.4)	27.7 (4.2)
Pentecostal	69.7 (11.7)	41.0 (7.8)	28.7 (4.8)
SDA	66.1 (11.3)	39.8 (8.8)	26.3 (4.6)
*Cadre*			
Consultant	80.0 (7.8)*	49.4 (3.7)*	30.6 (4.3)*
Medical officer	75.7 (9.4)	45.6 (6.2)	30.1 (5.2)
Clinical officer	68.2 (9.0)	41.4 (6.3)	26.8 (4.5)
Reg trained nurse	68.2 (12.4)	41.0 (8.5)	27.2 (5.0)
Enrolled nurse	64.7 (12.2)	38.1 (9.1)	26.6 (4.0)
Pharmacist	66.6 (8.8)	39.1 (6.3)	27.5 (3.4)
Radiographer	66.8 (5.0)	40.6 (4.7)	26.2 (1.1)
Laboratory technologist	70.1 (9.8)	41.9 (6.3)	28.2 (4.4)
Other	66.5 (10.9)	39.7 (7.2)	26.8 (4.9)
*Department*			
Emergency	70.5 (10.3)	42.8 (7.5)	27.7 (4.7)
Outpatient	67.6 (10.1)	40.4 (7.1)	27.2 (4.5)
Inpatient	67.6 (13.1)	40.1 (9.3)	27.5 (4.8)
Investigative dept	69.4 (7.9)	42.1 (5.2)	27.3 (4.1)
Other	68.8 (11.8)	41.5 (7.7)	27.3 (4.8)

*Statistically significant at *p* < 0.05

There was no significant variation in levels of knowledge across public and private health facilities. There was, however, a significantly higher proportion (0.0469) of HCWs in private facilities with knowledge on what PPE to use in the fight against COVID-19 compared to health workers in public facilities. Knowledge on how to properly don and doff the full PPE for COVID-19 care was significantly higher (*p* value = 0.0132) in private facilities compared to public facilities (Table 5).

**Table 5:** Variations in HCW knowledge by health facility type

	Public (n = 297) (%)	*P* value	Private Not for Profit (n = 73) (%)	*P* value	Total (n = 370) (%)
I know the symptoms of COVID-19	95	1.0000	96	0.7162	95
I am aware there are behavioral risk factors for COVID-19 transmission	93	1.0000	94	0.7573	93
I am aware COVID-19 mortality is heightened if patient has other	92	1.0000	96	0.2316	92
I know which patient needs to be tested for COVID	87	0.4281	96	0.0669	89
I am aware there are behavioral risk factors for COVID-19 progression	87	0.6976	90	0.6271	88
I am sure of what PPE to use in the fight against COVID	85	0.7152	94	0.0469*	86
I am aware that there are demographic risk groups for COVID-19 transmission	81	0.5033	89	0.2019	83
I feel confident that I know detailed clinical information about COVID-19	81	0.7409	86	0.4099	82
I am aware that there are demographic risk groups for COVID-19 mortality	78	0.5280	88	0.1094	80
I know there are some myths about causes and prevention	78	1.0000	75	0.5752	78
I know there are some falsehoods about causes, prevention and treatment of COVID-19	76	1.0000	75	0.8554	76
I know our hospital’s criteria for admission	75	1.0000	77	0.7175	75
I understand the terminology of the virus causing COVID-19	72	0.5629	81	0.2064	74
I know there are some myths about causes, prevention and treatment of COVID-19	73	0.7712	76	0.7210	74
I fell I am ready to manage COVID as a health care worker	67	0.5820	79	0.0868	69
I’m comfortable with the case definition of COVID in use in Uganda	69	0.6280	57	0.1012	67
I understand the type of testing necessary for COVID	65	0.8115	72	0.3197	66
I know how to properly don and doff the full PPE for COVID care	61	0.4816	79	0.0132*	64
I am aware of the treatment options for COVID-19	57	0.8193	53	0.6378	56
I have a good understanding of the pathophysiology of COVID-19	55	1.0000	54	0.8755	55
I know well how to administer appropriate levels of oxygen for COVID patients	42	1.0000	41	0.8744	42
If I need to intubate a patient, I understand the recommendations around NIPPV and protective gear	40	0.6418	30	0.1954	38
I know the criteria for intubation of COVID patients	38	0.4791	26	0.1370	35

Data from the qualitative arm of the study provides some insight and triangulation for quantitative results. First, there was a general understanding of COVID-19 among HCWs, particularly on its signs and symptoms. In some geographical areas training and strengthening the capacity of HCWs for case management had been conducted, particularly by health implementing partner agencies in refugee settlements:

*We have a team comprising of healthworkers who have been trained on COVID-19 case management… these healthworkers have been subjected to various, about three, simulations for COVID-19 case management. So during simulation exercises we bring in real life scenarios of an actual case of COVID-19 just to build their capacity, and make sure that they are ready to respond incase we get a confirmed case in the settlement here… and then it is mandatory for all the healthworkers to have basic knowledge. So I would say all of them have been trained, but some have been given further guidance with simulation exercises* (Implementing Partner, Settlement #1, Region 2)

*The moment we heard that its COVID our bosses immediately arranged trainings for us and also equipped us with the essential supplies like more PPEs to go along with that knowledge. Atleast now we know something although we are continuing to learn about this strange disease. Also, the Ministry of Health guidelines are helping us all* [HCW 1, Private Health Facility, Region 3]

However, findings in some public facilities showed that COVID-specific training had not yet taken place, although some information had been shared to increase HCW knowledge and awareness:

*Up to now we have not seen anyone from the district or Ministry* [of Health] *coming to train us on COVID. Maybe they will come with time… we received some of these posters on the disease and are using that information and our training to try and protect ourselves and our clients…we are improvising but it’s tough* (HCW 2, Public Health Facility, Region 2)

This was confirmed by data from the national level which also explained the government’s targeted and phased approach to strengthening health facility capacity and preparedness. The focus was particularly at referral points where most COVID-19 cases were going to end up, as opposed to spreading thin by attempting to simultaneously equip all health facilities at different levels:

*Part of the reason we locked down was to prepare care capacity and in preparing care capacity, we made sure that every district hospital has a COVID-19 care capacity… and a specialized COVID-19 team. We have moved around and trained, we made sure that regional referral hospitals have capacity… The model is such that the very severe cases of COVID-19 are supposed to be managed at the regional referrals* [hospitals]. *The district hospitals are supposed to manage the minor cases; and in this case because we have few cases and all the cases go to the regional referral, we have not covered all the hospitals yet. We have not gotten there yet but will get there. Because COVID-19 one day it’s going to become like malaria and the patients will go to any healthcare centre and be treated…But for now we make sure that the plan is expanded very well and it will come to reach to the point where we are going slowly* (KII, Policymaker)

In agreement with some of the quantitative findings on training HCWs and resulting knowledge, implementing partners and other stakeholders reported constraints with training materials and content, particularly in the early phase of COVID-19. They also highlighted the limitations that short training intervals have on the extent to which they can strengthen HCW capacity:

*All healthworkers in the settlement have been trained even though it is, I don’t know, maybe a one day’s training because the material on COVID is really not so much…and you have seen the trends, it is specifically the asymptomatic or mild cases so if a severe case happened I don’t think our healthworkers here can handle. They will immediately refer* (National-Settlement stakeholder 1)

Inspite of this training, there were some knowledge and skill gaps across most facilities, more especially at the lower-level health facilities:

*I won’t lie to you, most of us here know something about this COVID but not everything. It is a new disease and we are also just learning, just like everyone else. Ofcourse we may know abit more than the community but until we have proper training, we will just have to rely on our usual skills, the SOPs and the information as it keeps reaching us* (HCW 3, Public Health Facility, Region 1)

*We keep having our knowledge added on to slowly slowly but generally most healthworkers, myself included, do not feel very confident to handle a COVID case right now. So for me I suspect we shall refer* [to COVID management centres] *if we get one and have not been given extra training by that time* (HCW 4, Public Health Facility, Region 3)

## Practices

### Health care worker and facility practices regarding COVID-19

Study results show healthworker and facility practices regarding COVID-19 to be less than optimal in several regards. Between 5 and 30% strongly disagreed/disagreed that practices to ensure patient safety from transmission (e.g. disinfection, social distancing and ventilation) are adhered to or in place. Between 11 and 55% strongly disagreed/disagreed that practices and precautions to ensure staff safety (e.g. mapping out risk and hazards, staff safety and health contingency plans, non-critical staff working at home or shift working) were being adhered to. Over 30% strongly disagreed/disagreed that practices to improve communication (e.g. having a person assigned for the purpose) were in existence at their facility. Table 6 illustrates this further:

**Table 6:** COVID-19 practices

HCW/facility practices	Strongly disagree		Disagree		Agree		Strongly agree	
	n	%	n	%	n	%	n	%
Patients, visitors aware of COVID-19 prevention practices	23	6.2	81	21.9	194	52.4	69	18.7
HCWs aware of COVID-19 prevention practices	5	1.4	24	6.5	188	50.8	150	40.5
HCWs apply standard precautions for all patients	18	4.9	89	24.1	173	46.8	83	22.4
Droplets and contact precautions recommended for febrile, coughing patients	22	5.9	57	15.4	181	48.9	102	27.6
Patients should be placed in adequately ventilated rooms	3	0.8	20	5.4	164	44.3	174	47.0
One-meter distance between beds ensured for all patients	35	9.5	65	17.6	154	41.6	111	30.0
Hospital has an active COVID-19 taskforce	16	4.3	47	12.7	155	41.9	141	38.1
Hospital has written COVID-19 preparedness plans	36	9.7	95	25.7	146	39.5	87	23.5
People are assigned to communicate status and impact of COVID-19 to staff	40	10.8	79	21.4	165	44.6	85	23.0
People are assigned to communicate status and impact of COVID-19 with authorities	37	10.0	88	23.8	160	43.2	82	22.2
People are assigned to communicate with patients about COVID-19	29	7.8	101	27.3	172	46.5	67	18.1
Facility has mapped the COVID-19 risks/hazards for all work points	26	7.0	117	31.6	165	44.6	59	15.9
Staff safety is included in contingency plans	48	13.0	100	27.0	168	45.4	51	13.8
Non-critical staff asked to work from home	103	27.8	101	27.3	112	30.3	52	14.1
Shifts introduced to avoid HCW concentrations	82	22.2	93	25.1	125	33.8	69	18.7
Facility has a COVID-19 M&E mechanism	51	13.8	76	20.5	179	48.4	63	17.0
Facility trained management, workers on COVID-19 management	25	6.8	46	12.4	185	50.0	111	30.0
Staff assisted to minimize direct contact with customers, ensure personal hygiene	16	4.3	29	7.8	190	51.4	134	36.2
Workers in direct contact with clients have PPEs	27	7.3	48	13.0	182	49.2	111	30.0
Risk of COVID-19 in travel has been assessed	48	13.0	70	18.9	179	48.4	69	18.7
Facility maintains regular communication with workers	50	13.5	76	20.5	166	44.9	76	20.5
HCWs assisted to manage any emerging psychosocial risks	44	11.9	86	23.2	179	48.4	59	15.9
Virtual meetings preferred to Face-to-face meetings	59	15.9	102	27.6	132	35.7	72	19.5
Hand washing and/or sanitization culture promoted	9	2.4	19	5.1	151	40.8	188	50.8
Surfaces regularly wiped with disinfectants	16	4.3	60	16.2	161	43.5	131	35.4
Workspace ventilation is improved	28	7.6	86	23.2	166	44.9	87	23.5
Good respiratory hygiene promoted and communicated	7	1.9	33	8.9	167	45.1	161	43.5
Social distancing is promoted in congregate settings	27	7.3	58	15.7	157	42.4	127	34.3
HCWs suspected COVID-19 encouraged to self-isolate	22	5.9	62	16.8	148	40.0	135	36.5
HCWs informed what to do when they suspect to have COVID-19	13	3.5	41	11.1	182	49.2	131	35.4
Facility has planned disinfecting areas	14	3.8	43	11.6	177	47.8	133	35.9

Using Bloom’s cut-off points, there were high-level practices among HCWs on placing patients in adequately ventilated rooms (94%), respiratory, hand hygiene and prevention of healthcare-associated infections (92%); avoiding face-to-face meetings and giving preference to phone calls, email or virtual meetings (92%); improved ventilation around workspaces (89%) and training of facility management, workers or their representatives on management of COVID at the facility (88%).Workers with suspected symptoms of COVID-19 were encouraged not to come to the workplace and follow available guidance (85%). Additionally, staff were informed on what to do when they suspect to have COVID-19 (84%). The health facilities had an active taskforce to manage COVID-19 (82%); the facility had a monitoring and evaluation mechanism of the COVID-19 prevention strategies and plans (81%). Staff had been assisted to minimize the direct contact with customers and ensure personal hygiene practices such as hand washing and use of hand sanitizers (80%).

There were average-level practices on hand washing at the facility (79%). Droplets and contact precautions were recommended for febrile and coughing patients (78%), social distancing was being promoted in congregate settings where clients gather (77%), good respiratory (e.g. face mask) hygiene was promoted and communicated (77%) and the one- meter distance between beds ensured regardless of whether patients are suspected of having COVID-19 (73%). Patients and visitors were aware of respiratory and hand hygiene and prevention of healthcare-associated infections (72%); while HCWs were applying standard precautions for all patients (71%). Surfaces (e.g. desks and workstations, doorknobs, telephones keyboards and working objects) were regularly wiped with disinfectant (69%). HCWs who came in direct contact with clients had been provided with personal protective equipment (68%); health facility had written COVID-19 preparedness plans accessible to all staff (68%) and a person had been assigned responsibility for communications with staff, regarding the status and impact of COVID-19 (66%). The risk of COVID-19 in travel had been assessed with non-essential travel avoided (66%), shifts introduced to avoid large concentrations of workers in the facilities (66%) and a person had been assigned responsibility for communication with health authorities regarding the status and impact of COVID-19 (65%). The facility maintained regular communication with workers and their representatives, including over the internet, or when not possible, over the phone (65%); a person had been assigned responsibility for communications with patients, and their families regarding COVID-19 (61%). The facility had mapped the COVID risks/hazards of all work points and covering all jobs (60%).

**Fig. 2:**
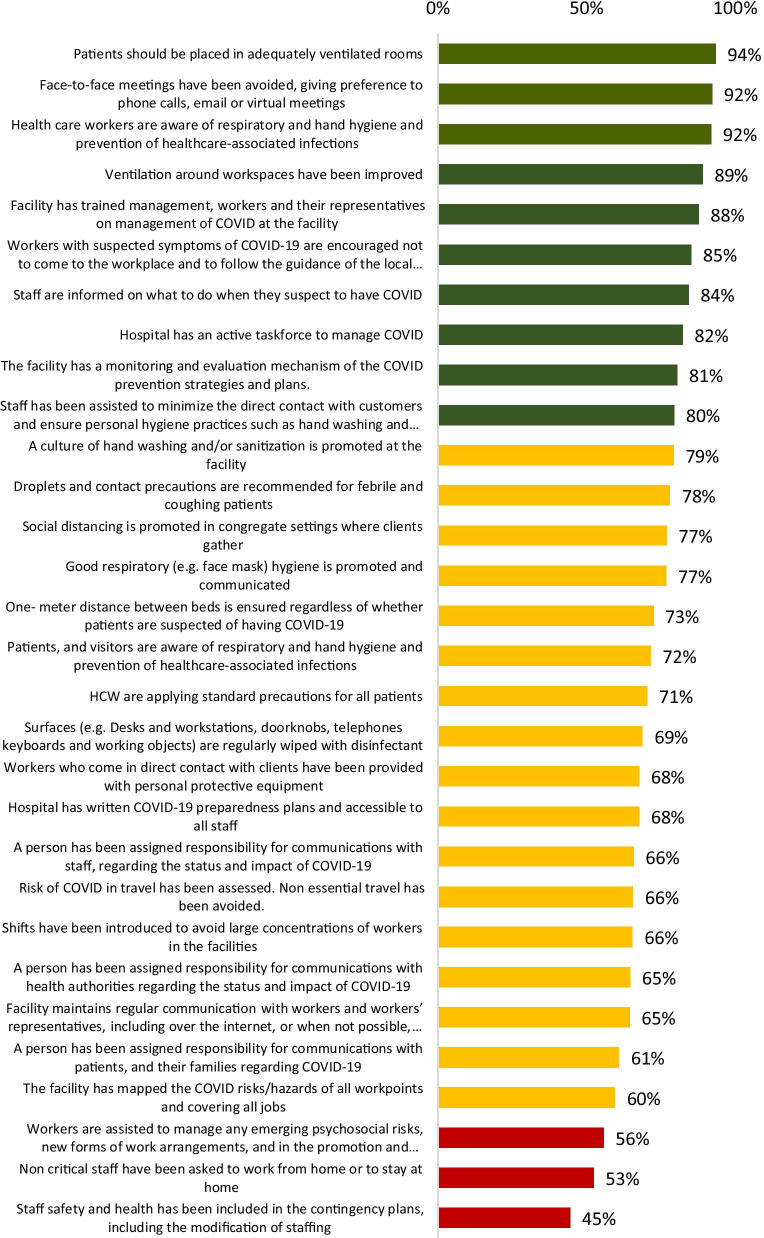
COVID-19 related practices among HCWs

There were low-level practices on assistance of workers to manage any emerging psychosocial risks, new forms of work arrangements, and in the promotion or maintenance of healthy lifestyles. Non-critical staff had been asked to work from home or to stay at home (53%) while staff safety and health had been included in the contingency plans, including the modification of staffing (45%). This information is shown in Fig. 2 below:

HCWs in the South Western region reported significantly higher level practices (*p* value = 0.0157) in avoiding non-essential travel as compared to the other regions. There was also a significant proportion (*p* value = 0.0312) of HCWs reporting that a person had been assigned responsibility for communications with health authorities regarding the status and impact of COVID-19 in the South West compared to other regions. HCWs in private health facilities reported relatively high-level practices compared to their counterparts in public facilities (Table 7).

**Table 7:** Variations in health worker practices by region represented

	Central (n = 171) (%)	*P* value	West Nile (n = 69) (%)	*P* value	South Western (n = 35) (%)	*P* value	Northern (n = 95) (%)	*P* value	Total (n = 370) (%)
Patients should be placed in adequately ventilated rooms	90	0.0959	97	0.3173	97	0.4664	97	0.2485	94
Face-to-face meetings have been avoided, giving preference to phone calls, email or virtual meetings	90	0.4416	91	0.7808	97	0.2857	96	0.1785	92
Health care workers are aware of respiratory and hand hygiene and prevention of healthcare-associated infections	88	0.1361	96	0.2436	97	0.2857	95	0.3192	92
Ventilation around workspaces have been improved	85	0.1877	96	0.0742	94	0.3583	89	1.0000	89
Facility has trained management, workers and their representatives on management of COVID at the facility	82	0.0609	91	0.4743	94	0.2878	94	0.0928	88
Workers with suspected symptoms of COVID-19 are encouraged not to come to the workplace and to follow the guidance of the local authorities	87	0.5379	87	0.6669	91	0.3356	78	0.1009	85
Staff are informed on what to do when they suspect to have COVID	86	0.5491	77	0.1567	89	0.4360	86	0.6321	84
Hospital has an active taskforce to manage COVID	83	0.777	74	0.1223	74	0.2465	91	0.0337	82
The facility has a monitoring and evaluation mechanism of the COVID prevention strategies and plans	74	0.0643	84	0.5564	91	0.1427	87	0.1729	81
Staff has been assisted to minimize the direct contact with customers and ensure personal hygiene practices such as hand washing and use of hand sanitizers	81	0.7858	75	0.3479	80	1.0000	80	1.0000	80
A culture of hand washing and/or sanitization is promoted at the facility	82	0.4182	75	0.4593	80	0.8896	77	0.6720	79
Droplets and contact precautions are recommended for febrile and coughing patients	76	0.6056	76	0.7144	79	0.8914	83	0.2862	78
Social distancing is promoted in congregate settings where clients gather	79	0.6041	72	0.371	89	0.1016	73	0.4148	77
Good respiratory (e.g. face mask) hygiene is promoted and communicated	78	0.7964	71	0.2842	86	0.2215	76	0.8370	77
One- meter distance between beds is ensured regardless of whether patients are suspected of having COVID-19	70	0.4701	78	0.3865	77	0.6094	72	0.8453	73
Patients, and visitors are aware of respiratory and hand hygiene and prevention of healthcare-associated infections	69	0.4748	72	1.000	86	0.0742	71	0.8470	72
HCW are applying standard precautions for all patients	67	0.3468	73	0.7362	77	0.4528	73	0.7007	71
Surfaces (e.g. Desks and workstations, doorknobs, telephones keyboards and working objects) are regularly wiped with disinfectant	74	0.2358	51	0.0038*	83	0.0838	68	0.8513	69
Workers who come in direct contact with clients have been provided with personal protective equipment	71	0.4836	66	0.7446	71	0.7151	63	0.3558	68
Hospital has written COVID-19 preparedness plans and accessible to all staff	63	0.2527	69	0.8701	80	0.1427	72	0.4533	68
A person has been assigned responsibility for communications with staff, regarding the status and impact of COVID-19	58	0.0726	68	0.7472	77	0.1866	74	0.1375	66
Risk of COVID in travel has been assessed. Non essential travel has been avoided	63	0.4966	66	1.0000	86	0.0157*	63	0.5839	66
Shifts have been introduced to avoid large concentrations of workers in the facilities	61	0.2592	63	0.6307	71	0.5499	74	0.1375	66
A person has been assigned responsibility for communications with health authorities regarding the status and impact of COVID-19	58	0.1177	70	0.4221	83	0.0312*	66	0.8553	65
Facility maintains regular communication with workers and workers’ representatives, including over the internet, or when not possible, over the phone	65	1.0000	63	0.7499	69	0.6350	64	0.8557	65
A person has been assigned responsibility for communications with patients, and their families regarding COVID-19	58	0.5081	52	0.1624	74	0.1301	68	0.2094	61
The facility has mapped the COVID risks/hazards of all workpoints and covering all jobs	63	0.5064	51	0.164	66	0.4882	58	0.7234	60
Workers are assisted to manage any emerging psychosocial risks, new forms of work arrangements, and in the promotion and maintenance of healthy lifestyles	62	0.1892	50	0.3582	54	0.8201	49	0.2221	56
Non-critical staff have been asked to work from home or to stay at home	49	0.3871	52	0.8787	54	0.9099	58	0.3836	53
Staff safety and health has been included in the contingency plans, including the modification of staffing	49	0.3861	38	0.2826	57	0.1739	36	0.1145	45

HCW ratings of their practices varied geographically. At district level, HCWs in Gulu and Kyegegwa rated their practices significantly higher than HCWs in other districts on average. In terms of facility variations HCWs at HCIII rated their overall staffing, communication, risk mapping and monitoring-related practices significantly higher than HCWs at other levels on average. There were no significant variations in HCWs practices at department or cadre level. Marital status was significantly associated with HCWs self-ratings although the pattern of variation was inconsistent across the different facets of practice. There were no variations in HCW practices by gender, religion and education (Table 8).

**Table 8:** Variations in HCWs practices by socio-demographic and facility factors

Characteristic	Overall practice index (31–124) Mean (SD)	Patient safety index (7–28) Mean (SD)	Staff safety index (12–48) Mean (SD)	Communication, risk mapping and monitoring index (11–44) Mean (SD)
*District*				
Adjumani	82.5 (15.3)*	20.6(5.0)*	31.7 (6.9)*	30.2 (5.8)*
Gulu	96.0 (12.3)	22.4(4.3)	37.7 (5.5)	35.9 (4.7)
Kabarole	82.4 (15.0)	20.7 (3.4)	31.7 (6.7)	30.1 (6.7)
Kampala	85.6 (13.5)	20.9 (3.2)	33.8 (6.0)	30.9 (5.7)
Kyegegwa	91.6 (16.8)	22.2 (3.7)	36.2 (7.4)	33.2 (6.8)
Mubende	87.5 (15.5)	21.1 (3.5)	34.6 (6.7)	31.7 (6.9)
*Health facility level*				
Region Referral hospital	86.8(13.9)*	21.1 (3.4)	33.8 (6.3)*	31.9 (6.0)*
General hospital	82.2(14.8)	20.8 (4.4)	31.6 (6.5)	29.8 (6.0)
HC IV	83.6(17.8)	20.6 (4.4)	33.4 (7.1)	29.6 (7.2)
HC III	98.3 (15.3)	23.1 (5.0)	39.4 (6.9)	35.8 (5.4)
HC II	86.3(6.9)	21.0 (2.4)	34.6 (4.0)	30.8 (3.4)
Clinic	95.4(10.1)	22.1 (2.6)	38.2 (5.4)	35.1 (4.5)
*Gender*				
Male	86.0 (14.9)	20.9 (3.9)	33.7 (6.8)	31.4 (6.2)
Female	87.2 (15.2)	21.3 (3.8)	34.1 (6.6)	31.7 (6.4)
*Education*				
Certificate	88.8 (15.5)	22.0 (3.9)	34.8 (6.9)	32.0 (6.9)
Diploma	85.4 (15.5)	20.7 (4.0)	33.3 (6.8)	31.3 (6.3)
Bachelors’ degree	86.7 (15.0)	21.0 (3.4)	34.1 (6.1)	31.6 (6.2)
*Marital status*				
Single	89.7 (14.9)*	21.9 (3.3)*	35.0 (6.9)	32.8 (6.0)*
Married	85.1 (14.6)	20.9 (3.8)	33.3 (6.4)	30.9 (6.2)
Divorced/separated	86.2 (16.4)	18.6 (7.0)	34.9 (7.5)	32.7 (6.4)
Widowed	93.5 (32.4)	20.3 (9.1)	38.5 (11.2)	34.8 (12.6)
*Religion*				
Catholic	88.8 (15.7)	21.6 (4.1)	34.8 (6.8)	32.5 (6.4)
Protestant	85.0 (14.2)	20.8 (3.4)	33.3 (6.5)	30.9 (5.9)
Muslim	86.8 (14.9)	20.8 (4.0)	34.0 (6.5)	32.0 (5.9)
Pentecostal	86.1 (14.9)	21.3 (3.5)	33.9 (6.8)	30.8 (6.5)
SDA	79.1 (13.7)	19.1 (5.3)	30.3 (6.9)	29.2 (6.3)
*Cadre*				
Consultant	96.9 (14.1)	22.4 (2.5)	37.4 (7.0)	37.0 (5.6)
Medical officer	85.0 (14.6)	20.4 (4.6)	33.3 (6.4)	31.3 (6.2)
Clinical officer	83.6 (16.0)	20.3 (4.6)	33.0 (7.1)	30.3 (6.9)
Reg trained nurse	86.5 (13.3)	20.7 (3.1)	34.0 (5.9)	31.8 (5.8)
Enrolled nurse	86.4 (17.2)	21.5 (4.5)	33.7 (7.6)	31.1 (6.8)
Pharmacist	87.0 (12.5)	21.3 (2.8)	34.2 (5.5)	31.5 (6.2)
Radiographer	78.6 (7.9)	20.0 (2.1)	30.2 (4.1)	28.4 (2.9)
Laboratory technologist	87.4 (12.8)	21.4 (3.2)	34.6 (5.8)	31.4 (5.4)
Other	89.3 (16.0)	21.9 (3.8)	34.6 (7.4)	32.8 (6.2)
*Department*				
Emergency	87.2 (14.7)	19.9 (2.4)	33.9 (8.4)	33.4 (6.0)
Outpatient	87.7 (14.3)	21.4 (4.1)	34.6 (6.2)	31.8 (5.8)
Inpatient	84.5 (16.1)	20.9 (3.7)	32.7 (6.9)	30.8 (6.9)
Investigative dept	89.1 (15.0)	21.5 (3.4)	35.3 (6.0)	32.3 (6.6)
Other	87.9 (14.7)	21.3 (3.7)	34.6 (7.0)	32.1 (6.0)

*Statistically significant at *p* < 0.05

### Work environment

**Fig. 3:**
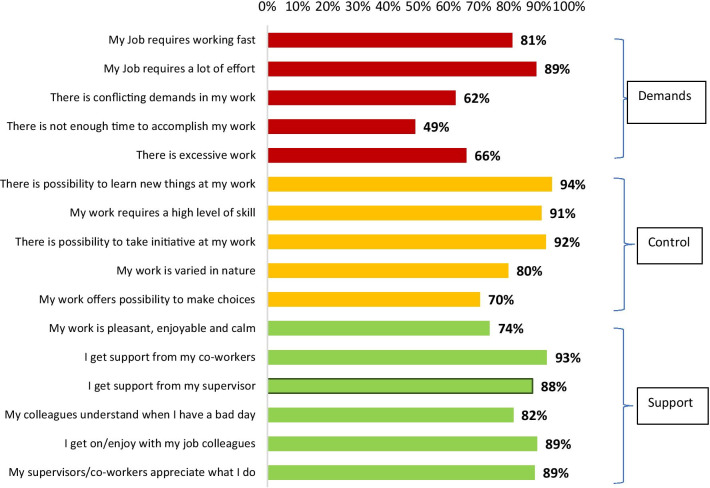
HCW perceptions on their work environment in relation to COVID-19

HCWs reported increased demands in their work environment in the COVID-19 pandemic era. 81% indicated that their job requires them to work fast with a lot of effort (89%), presents conflicting demands (62%), excessive work (66%) and lack of adequate time to accomplish work (49%). However, they also reported increased control in the work environment indicated by a high proportion of HCWs reporting the need to learn new things at work (94%), high level of skill (91%), the need to take initiative at work (92%), variation in the nature of work (80%) and possibility of making choices at work (70%). Inspite of their changing work environment, HCWs reported that they get adequate support in their work. 74% indicated that their work is enjoyable, pleasant and calm, with support from co-workers (93%) and supervisors (88%) as well as appreciation from supervisor (89%). The information is shown in Fig. 3.

There was no significant variation in HCW perceptions of their work environment across all regions. There was, however, a significantly lower proportion (*p* value = 0.0387) of HCWs in West Nile reporting that the job requires them to work fast compared to the other regions. A significantly higher proportion (*p* value = 0.0257) of HCWs in the South Western region reported that the job requires them to work fast compared to the other regions. There was no significant variation in the perception of work environment among HCWs across public and private health facilities, gender or education level.

Qualitative findings on HCW preparedness and frontline practices gave a somewhat different picture in terms of workloads and work environment. While HCW at referral health facilities reported increased workloads, those at lower level facilities reported reduced patient numbers to attend to in the pandemic era. Some of the explanatory factors for low workloads include COVID-19 movement restrictions, the health system’s referral pathway, decongesting high-volume facilities through task shifting or innovative community-based mechanisms and community reluctance to utilize health services—even for suspected COVID-19 cases, due to fear of related stigma and/or isolation:

*The stigma attached to respiratory illnesses would consider anyone suffering from respiratory illness to be suffering from COVID so it makes people to fear seeking medical attention… these days people who are coughing first struggle with their cough from home before coming here* (HCW, In-Settlement Facility 3, Region 1)

*Ever since March when the President said “no movement”, we have much fewer patients… we nolonger do community dialogues, public health talks and sensitizations all because of COVID. To try and adapt we are using community-based surveillance for case detection and management through ICCM programs like malaria so that people who are badly off can be managed at that level which reduces the turnover at the facility* (HCW 5, Region 2)

Health care workers, particularly those at in-settlement health facilities, confirmed being prepared to manage COVID-19 cases:

*Our involvement begins from the settlement level to the district level because even as a facility we have a rapid response team. Some members of our facility got training to respond to any case* (HCW, In-Settlement Health Facility 1, Region 3)

*We are always prepared for epidemics so our level of preparation is somewhere. We have the facilities like the treatment unit, isolation unit within, training of the team and mobilisation is easier and ofcourse we ensure adherence to the SOPs by all staff* (HCW, In-Settlement Health Facility 3, Region 1)

This study found one of the biggest challenges to HCW capacity to mostly be infrastructure and supply-related. Although a number of HCWs at public facilities reported not receiving any or adequate training or capacity building sessions on COVID-19 care, they reported “winging it” or improvising in terms of practice. However, all of them reported struggling to deliver quality healthcare with limited infrastructural adjustments and/or supplies:

*I am equipped with information but for utilities like PPEs I am not well equipped* (HCW 6, Public Health Facility, Region 4)

*I don’t think there is a country that has everything, but supplies are a big problem. I personally move with gloves in my bag because we don’t have any in the health centre. Last month we only had two boxes of gloves as the whole health centre… 50 pairs in each, that is 100 pairs of gloves…how are we meant to work with these gloves for 60 days?* (HCW 7, Public Health Facility, Region 3)

*This situation has come with the demand for more human resource and space… also inadequate logistics because stockouts are the order of the day. That includes gloves, handwashing facilities, facemasks and others* (HCW, Public Health Facility, Region 4)

*Equipment is rare as you can be equipped today and it all runs out the following day because most of utilities for COVID-19 are consumables and disposables so we use them on a daily basis. Support in terms of PPEs is supposed to be a continuous exercise but we don’t always have so I don’t feel I am adequately equipped to manage* (HCW, Public Health Facility, Region 2)

## Discussion and conclusions

This study assessed HCW perceptions about their knowledge and awareness of COVID-19. We found knowledge to be generally low in some areas. Specifically, knowledge regarding symptoms, criteria for admission, treatment options, protection and safety promotion was rated low by a significant number of HCWs. This could be attributed to the study timing where data was collected at the beginning stages of the pandemic with only few COVID-19 cases confirmed in the study setting at the time. The limited knowledge on COVID-19 among HCWs can also partly be explained by a general lack of patients, training and ready guidelines on how to handle a new pandemic. This challenge was not only reported by this study’s participants but also several other studies across the world [38, 56, 57] which confirm a paucity of information and progressive lessons in COVID-19 detection, prevention and management. Clinical guidelines and other related directives began to take shape after pandemic onset and research conducted in early phases was bound to find a general gap in knowledge and practice skills. This gap was exacerbated by the initial comparatively low transmission rates and almost no confirmed COVID-19 cases at most health facilities for an extended period of time. Uganda managed to hold off pandemic mortality for some time—its first COVID-19 case was confirmed on 21st March 2020 and first death registered four months later on 23rd July 2020. In addition, Uganda’s health system design and national response to COVID-19 required prompt referral to higher-level facilities as opposed to managing cases at all levels [58].

Geographical, regional and district variations in HCW knowledge, attitude and practices were observed, with the South-western (Kyegegwa) and Northern region (Gulu) scoring highest. Several factors could account for these differences. First, Kyegegwa is a direct refugee-hosting district with Kyaka II refugee settlement and has a resident implementing partner (IP) exclusively handling refugee health matters within the settlement, although it also serves the surrounding host communities. Humanitarian health partners across all settlements actively trained and supported COVID-19 management in their respective units [28, 59] partly because they were not constrained or directly under government but supporting the national refugee response. Moreover, the South Western and Northern region had an epidemic management history with response preparedness planning and training following the 2018–2021 Ebola outbreak. This previous experience in managing epidemics also explains the reported higher knowledge levels.

The dynamism and complexity of Uganda’s health system design has been highlighted in previous studies [54]. System strengthening led by development and implementing partners in refugee-hosting areas could partly explain why some lower-level HCIIIs reported high level knowledge compared to HCIVs or district hospitals. This is because most in-settlement health facilities are at HCIII level, which was not initially prioritized for full scale COVID-19 training and equipping in the government’s phased approach to system strengthening. This would also be a plausible explanation for the underlying KAP variations by health facility ownership (i.e. public vs. private, including profit and not-for-profit private facilities) and community care-seeking behaviour which tends to prefer private over public health facilities, especially for emergencies [60–63]. Gulu as the regional referral lead, where West Nile [Adjumani district] health facilities also referred COVID-19 cases, would have higher knowledge levels and stronger system capacity. Moreover, Gulu has been at the forefront and has history of managing epidemics such as Ebola [64, 65] so the infrastructure and experience is in place. A number of studies have indeed supported this notion of system preparedness and resilience following an epidemic [66–68]. We recommend that, due to the evolving nature and trajectory of COVID-19, stronger communities of practice are supported for cross-learning and good practices adopted from more experienced health facilities with flexibility for amendment as new evidence unfolds.

Regional variations on knowledge e.g. on infodemics, which show HCWs in the central region to have higher knowledge than their counterparts, could be due to better information access on COVID-19. Kampala, also the capital city and central business area, was and remains epicenter of the national COVID-19 response. Initially access to information was mostly available via radio, television and print media mostly accessible in urban settings [69]. We recommend further engagement of all HCWs and ongoing information sharing, including through their professional and regulatory bodies.

In all aspects of knowledge, HCWs with atleast a bachelors’ degree education exhibited higher knowledge than peers with a diploma or certificate level education. There is nothing surprising there as the evidence shows direct linkages between HCW education level, regulation, supervision, competence to understand or perform deliver services and improved clinical outcomes in healthcare [70, 71]. However, training and competence variations also highlight the need for comprehensive, systematic, tailored and refresher training for all HCWs with clear outcomes—both for learning and service delivery at the point of care. This is especially needed in resource-limited settings; in light of limited health workforce and fragile health systems which also require task shifting, ongoing supervision and supportive mechanisms [48, 52]. The knowledge, skill, and experience of HCWs remain critical inputs for system capacity and resilience [42] during a pandemic and its aftermath. In light of the low human resource capacity, need for task shifting and ongoing capacity building interventions, we recommend a follow-on study that assesses post-training and in-service knowledge and skills of different cadres of health providers 18 or more months into the pandemic, possibly following up the same cohort.

Capacity building methods for healthworkers have a bearing on knowledge retention and application [72, 73]. This study’s findings show that the majority of HCWs did not have first-hand experience in managing COVID-19 cases, although some had undergone simulation training in preparation and nearly all HCWs could apply basic clinical knowledge to triage, screen, isolate and refer patients. The evidence shows on-site mentorship and support at the point of care, managing actual cases, to be more beneficial and impactful if certain conditions are met [74–76]. This is for not only service delivery outcomes but also knowledge retention and translation or application among HCWs [75, 76]. HCW and facility practices to effectively manage COVID-19 are constrained by individual factors (e.g. education level, residence, location of work station, hygiene promotion or social distance management) and facility-related factors (e.g. poor information/communication, lack of safety plans, risk monitoring or work environmental threats).The need for infrastructural preparedness, responsive and resilient health systems cannot be overemphasized [29, 68]. We recommend practical, bespoke and urgent strengthening of system capacity at all levels, especially in light of surging community transmission, a limited health workforce and congested higher-level facilities. This will partly require optimally harnessing the benefits of task-shifting in addition to strengthening cross-site mentorship, learning, coordination and the referral pathway. Robust processes will entail more on-site learning, leveraging technology and the use of data in decisionmaking.

In light of Uganda’s fragile health system and limited capacity at most health facilities, there is need to empower communities and patients to prevent and self-manage certain conditions, while emphasising health literacy and telemedicine. Strengthening community-based surveillance and ensuring functional health systems for disease prevention and management is critical in the era of COVID-19, especially for resource-limited settings. There is need to equip community members, leaders, village health teams (VHTs) and lower- level facilities with capacity to respond effectively. In October 2020, and against the backdrop of surging community transmissions, Uganda launched a Community Health Engagement Strategy (CES) with key pillars and a promise to invest the required resources needed for its activation and functionality [77]. Collaboratively engaging local [political, administrative, technical, cultural and religious] leaders to take ownership and emphasise adherence to prevention measures will not only contribute significantly to health system resilience but also community agency, meaningful involvement beyond longstanding tokenism as well as stronger capacity to address the current and future pandemics.

### Limitations

The limitations of this study warrant some acknowledgement. First, this was a rapid assessment of the lived and implementation experiences in refugee settings regarding COVID-19 in specific areas including knowledge, awareness and risk behaviours. As such a cross-sectional study design was most appropriate. With cross sectional findings nonetheless, caution should be practiced in assuming causality. We can only firmly establish associations. The study was conducted in the earlier phase of Uganda’s first wave of COVID-19. Actual data collection was between September and October 2020 and a lot, including HCW knowledge or health facility capacity, has since changed. More importantly, this study relied on reported knowledge by study participants in lieu of administering knowledge assessments which could have created bias. These challenges notwithstanding, the study seems to be in line with our observations and hypotheses, also providing new insights for the effective management of COVID-19 among refugees in Uganda.

## Data Availability

All data generated or analysed during this study are included in this published article.

## Abbreviations

CES: Community Health Engagement Strategy
GDP: Gross Domestic Product
HC: Health Centre
HCWs: Health Care Workers
IDI: Indepth Interview
IPC: Infection Prevention and Control
KAP: Knowledge, Attitudes and Practices
KCCA: Kampala Capital City Authority
MakSPH-HDREC: Makerere University School of Public Health Higher Degrees Research Ethics Committee
MNHC: Maternal, Newborn and child health
MoH: Ministry of Health
NGOs: Non Governmental Organisations
NIPPV: Non Invasive Positive Pressure Ventilation
OPM: Office of the Prime Minister
PPE: Personal Protective Equipment
UNCST: Uganda National Council of Science and Technology
